# Comparison of swept-source versus spectral-domain optical coherence tomography angiography for detection of macular neovascularization

**DOI:** 10.1007/s00417-021-05229-6

**Published:** 2021-07-06

**Authors:** Anna Lentzsch, Laura Schöllhorn, Christel Schnorr, Robert Siggel, Sandra Liakopoulos

**Affiliations:** 1 Cologne Image Reading Center, Department of Ophthalmology, Faculty of Medicine and University Hospital Cologne, Cologne, Germany; 2 Department of Ophthalmology, Helios University Hospital Wuppertal, University of Witten‐Herdecke, Wuppertal, Germany; 3 Department of Ophthalmology, Goethe University, Frankfurt, Germany

**Keywords:** Optical coherence tomography angiography, Macular neovascularization, Swept-source OCTA, Spectral-domain OCTA

## Abstract

**Purpose:**

To compare swept-source (SS) versus spectral-domain (SD) optical coherence tomography angiography (OCTA) for the detection of macular neovascularization (MNV).

**Methods:**

In this prospective cohort study, 72 eyes of 54 patients with subretinal hyperreflective material (SHRM) and/or pigment epithelial detachment (PED) on OCT possibly corresponding to MNV in at least one eye were included. OCTA scans were acquired using two devices, the PLEX Elite 9000 SS-OCTA and the Spectralis SD-OCTA. Fluorescein angiography (FA) was used as reference. Two graders independently evaluated en face OCTA images using a preset slab as well as a manually modified slab, followed by a combination of en face and cross-sectional OCTA.

**Results:**

Sensitivity (specificity) for the automated slabs was 51.7% (93.0%) for SS-OCTA versus 58.6% (95.3%) for SD-OCTA. Manual modification of segmentation increased sensitivity to 79.3% for SS-OCTA but not for SD-OCTA (58.6%). The combination of en face OCTA with cross-sectional OCTA reached highest sensitivity values (SS-OCTA: 82.8%, SD-OCTA: 86.2%), and lowest number of cases with discrepancies between SS-OCTA and SD-OCTA (4.2%). Fleiss kappa as measure of concordance between FA, SS-OCTA, and SD-OCTA was 0.56 for the automated slabs, 0.60 for the manual slabs, and 0.73 (good agreement) for the combination of en face OCTA with cross-sectional OCTA. Concordance to FA was moderate for the automated slabs and good for manual slabs and combination with cross-sectional OCTA of both devices.

**Conclusion:**

Both devices reached comparable results regarding the detection of MNV on OCTA. Sensitivity for MNV detection and agreement between devices was best when evaluating a combination of en face and cross-sectional OCTA.


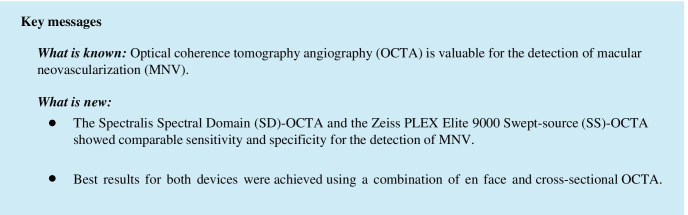


## Introduction

Optical coherence tomography angiography (OCTA) has become a valuable imaging tool, as it provides non-invasive, high-resolution, depth-resolved visualization of the retinal and choroidal microvasculature in situ [1, 2]. Three-dimensional images of vascular architecture are created via repeated OCT B-scans at the same position, and allow visualization of retinal and choroidal blood flow by detecting movement of erythrocytes within blood vessels [3, 4]. Flow information is either provided as en face view (slabs are defined by an inner and outer segmentation boundary), or as cross-sectional view with flow information displayed over OCTA B-scans [2, 5]. OCTA has been demonstrated to allow visualization of macular neovascularization (MNV) using automated as well as manually modified en face OCTA slabs [3, 6]. However, visualization of MNV on en face OCTA images may be affected by various artifacts and—in contrast to cross-sectional OCTA—depends on the correct position of segmentation lines [1].

Spectral-domain (SD) and swept-source (SS) OCTA devices are commercially available. OCTA devices vary regarding technical and software components, e.g. segmentation algorithms, preset slab definitions and other device-specific algorithms [2, 7]. The aim of this study was to compare the SS-OCTA device PLEX Elite 9000 (Carl Zeiss Meditec, Inc., USA) and the SD-OCTA device Spectralis (Heidelberg Engineering, Germany) regarding the detection of MNV in eyes with various chorioretinal diseases using preset and manually modified en face OCTA images, as well as using a combination with cross-sectional OCTA, blinded to other multimodal imaging modalities.

## Material and methods

This prospective study was conducted at the Department of Ophthalmology, University of Cologne, Germany in accordance with the ethical standards of the Declaration of Helsinki and approved by the institutional review board (register number 16–289). Written informed consent of all patients was obtained.

A total of 54 consecutive patients with various chorioretinal diseases and with subretinal hyperreflective material (SHRM) and/or pigment epithelial detachment (PED) on SD-OCT images possibly corresponding to a MNV in at least one eye were included. Collected patient data included age, gender, medical history and previous anti-vascular endothelial growth factor (VEGF) treatment.

### Image acquisition

OCTA images were obtained for all eyes with suspicion for MNV, as well as for fellow eyes if the patient agreed using both the SS-OCTA device PLEX Elite 900 (Carl Zeiss Meditec, Dublin, CA, USA) and the SD-OCTA device Spectralis (Heidelberg Engineering, Heidelberg, Germany). A scan pattern of 3 × 3 mm was chosen for the PLEX Elite SS-OCTA and correspondingly the 10° × 10° scan pattern for the Spectralis SD-OCTA to allow direct comparison.

The 3 × 3 mm PLEX Elite SS-OCTA scan consists of 300 B-scans repeated four times at each position resulting in a distance between section images of 10 µm. The 10° × 10° Spectralis SD-OCTA scan consists of 512 B-scans repeated 7 times at each position resulting in a distance between section images of ~ 6 µm.

The projection artifact removal (PAR) function was turned off. OCTA scans were centered on the fovea. When the area suspicious for MNV was located extrafoveal and was not captured on the central scan, an additional scan was focused on the MNV lesion. Scan position was identical for both devices.

Color fundus photography (Canon CX-1 digital camera, Canon, Tokyo, Japan), fluorescein angiography (FA) images and SD-OCT volume scans (Spectralis HRA + OCT, Heidelberg Engineering, Heidelberg, Germany) were collected as part of the clinical routine. Images were captured on the same day and imaging was performed with pupil dilation.

### Export of OCTA en face images

**Fig. 1:**
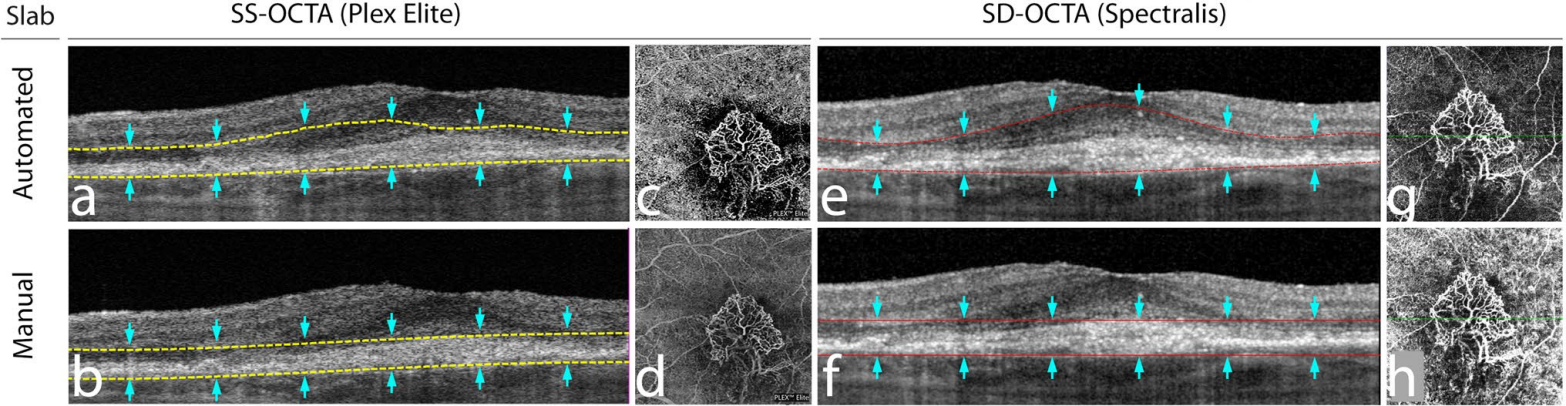
Segmentation of the automated and manually modified swept- source optical coherence tomography angiography (SS-OCTA) and spectral domain (SD)-OCTA en face slabs. The top row shows segmentation lines of the automated PLEX Elite SS-OCTA slab outer retina to choriocapillaris (ORCC) (**a**) and the Spectralis SD-OCTA avascular complex slab (**e**) as well as the corresponding OCTA en face images (**c** + **g**). The bottom row shows the segmentation of the manual slabs for SS-OCTA (**b**) and SD-OCTA (**f**) and the corresponding OCTA en face images (**d** + **h**). For the PLEX Elite SS-OCTA, the retinal pigment epithelium (RPE) fit layer was manually shifted and positioned anterior and posterior to any suspected macular neovascularization (MNV) (subretinal hyperreflective material (SHRM) and/or pigment epithelial detachment (PED)). For the Spectralis SD-OCTA, the transverse line was used with the same approach

Grading was performed using one automated slab for each device (the outer retina to choriocapillaris (ORCC) slab for the PLEX Elite SS-OCTA device and the avascular complex slab for the Spectralis SD-OCTA device), as well as one manual slab that was generated by adjustment of segmentation lines. To create the manual slab with the PLEX Elite SS-OCTA, the automatically provided RPE-fit layer was selected as the inner boundary of the slab and shifted anterior to any suspected MNV (SHRM and/or PED) (Fig. 1). The RPE-fit layer was also used as the outer boundary and shifted posterior to any suspected MNV (thus reaching the level of Bruch’s membrane or—in case the automatically generated layer did not run parallel to BM—posterior to BM).

For the manual slab of the Spectralis SD-OCTA, two transverse lines were selected as segmentation lines and shifted anterior and posterior to any SHRM and/or PED aiming to include the total area of a suspected MNV (Fig. 1).

An overview of the inner and outer slab boundaries is given in Table 1.

**Table 1:** Boundaries of the SS-OCTA and SD-OCTA preset and manual slabs

Device	En face slab	Inner segmentation line		Outer segmentation line	
		Name	Position	Name	Position
Automatically by the software provided slabs					
SS-OCTA PLEX Elite 9000	ORCC	OPL surface	The most posterior position of: •110 µm anterior to the RPE-fit* layer •30 µm anterior to the RPE band •200 µm posterior to the ILM	RPE-fit* + 37 µm	37 µm posterior to the presumed original position of the RPE band
SD-OCTA Spectralis	Avascular complex	OPL	At the level of the OPL	BM	At the level of BM
Manually modified slabs: manual adjustment of automated segmentation lines					
SS-OCTA PLEX Elite 9000	Custom	RPE-fit*	Anterior to any present SHRM and/or PED on all B-scans	RPE-fit*	At the level of/or posterior to Bruch’s membrane on all B-scans
SD-OCTA Spectralis	Custom	Transverse line	Anterior to any present SHRM and/or PED on all B-scans	Transverse line	At the level of/or posterior to Bruch’s membrane on all B-scans

*BM* Bruch’s membrane *ILM* Internal limiting membrane *OPL* Outer plexiform layer *ORCC* Outer retina to choriocapillaris *PED* Retinal pigment epithelial detachment *RPE* Retinal pigment epithelium *SHRM* Subretinal hyperreflective material

*The “RPE-fit” segmentation line is automatically positioned by the software at the presumed original position of the RPE. The RPE band is detected by the software and in areas with PEDs; the original position of the RPE is estimated based on surrounding healthy RPE. Bruch’s membrane is calculated to be positioned 29 µm posterior to the “RPE-fit” line (manufacturer’s information).

### Grading

Two independent masked graders (RS, CS) of the Cologne Image Reading Center and Laboratory (CIRCL) performed evaluation of all images. OCTA en face images were evaluated separately for each patient and imaging device. The graders were masked to all other imaging modalities and patient data. After evaluation of en face images alone, a combination of en face slabs and cross-sectional OCTA B-scans with flow information was used for grading. Discrepancies were discussed in an open adjudication. If no consensus was obtained, the final decision was made by the Reading Center Medical Director (SL).

MNV was defined on en face OCTA as a flow signal which represents a closed vascular network with capillary branching *or* a flow signal with vascular pattern within the expected foveal avascular zone not corresponding to the physiological retinal or choroidal vascularization. On cross-sectional OCTA, MNV was identified based on a flow signal within SHRM or a PED not representing projection artifacts of overlying retinal vessels or other artifacts such as motion artifacts.

MNV lesion subtypes evaluated on FA included predominantly classic MNV, minimally classic MNV, occult MNV and staining scar. In addition, retinal angiomatous proliferation (RAP) lesions were identified.

Grading options included “yes” if the graders were ≥ 90% certain that MNV is present, “questionable” if certainty ranged between 50 and 90%, and “no” in case graders were less than 50% certain. If severe artifacts were present precluding evaluation of MNV, images were graded as “cannot grade”. For statistical analysis, “questionable,” “no,” and “cannot grade” were combined.

### Statistical analysis

Statistical analysis was performed using SPSS (SPSS software version 25.0 for Mac, SPSS Inc. Chicago, IL, USA). The analysis included demographic data (age, gender) and clinical information (BCVA, diagnosis, history of anti-VEGF treatment). Sensitivity and specificity for MNV detection on OCTA was calculated using FA as the reference. Cases graded as “questionable” or “cannot grade” were grouped with “no” for the statistical analysis. The level of significance was set at *p* < 0.05. Cohen’s kappa statistic was used to calculate the concordance between FA and OCTA as well as between both OCTA devices and was classified according to Landis and Koch (> 0.80 represents “excellent” agreement, 0.61–0.80 “good” agreement, 0.41–0.60 “moderate” agreement, and < 0.40 “poor” agreement). Fleiss’ kappa statistic was used to calculate agreement of both devices and FA as ground truth.

## Results

### Study population

Seventy-two eyes of 54 consecutive patients were included. In 18 patients, both eyes were included. In 36 patients, OCTA images from both devices were available only from the eye with possible MNV. Demographic data and clinical characteristics of the study population are depicted in Table 2.

**Table 2:** Demographic data and underlying disease

Demographic data	
Number of eyes (*n*)	72
Number of patients (*n*)	54
Gender (male/female) (*n* (%))	29 (53.7%)/25 (46.3%)
Age (years) (mean ± SD (range))	62 ± 20 (19–91)
Prior anti-VEGF treatment (*n* (%))	16 (22.2%)
**Diagnosis on multimodal imaging (*n* (%))**	
AMD	28 (38.9%)
Chorioretinitis	9 (12.5%)
CSCR	7 (9.7%)
High myopia	5 (6.9%)
Pattern dystrophy	4 (5.6%)
Acquired vitelliform lesion	3 (4.1%)
Others	12 (16.7%)
Healthy eyes	4 (5.6%)

*AMD* age-related macular degeneration, *anti-VEGF* anti-vascular endothelial growth factor, *CSCR* central serous chorioretinopathy, *SD* standard deviation

In 29 eyes (40.3%), MNV was detected on FA. MNV lesion subtypes were classified as predominantly classic MNV in 14 eyes (37.8%), minimal classic MNV in 2 eyes (5.4%), and occult MNV in 7 eyes (18.9%). In 2 eyes with occult MNV (2.8%), a RAP was identified additionally. Six eyes (16.2%) showed a staining scar. In a total of 43 eyes (59.7%), no or only questionable MNV was detected on FA.

### Sensitivity and specificity for MNV detection

Grading results for SS-OCTA and SD-OCTA as well as discrepancies compared to FA and between OCTA devices are detailed in Table 3. Sensitivity and specificity values for MNV detection with OCTA are shown in Table 4.

**Table 3:** Grading results for SS-OCTA and SD-OCTA

	MNV present *n* (%)	No MNV present *n* (%)	Discrepancy to FA *n* (%)	Discrepancy between devices *n* (%)
FA	29 (40.3)	43 (59.7)	-	-
SS-OCTA PLEX Elite 9000				
ORCC slab	18 (25.0)	54 (75.0)	17 (23.6)	9 (12.5)
Manual slab	29 (40.3)	43 (59.7)	12 (16.7)	13 (18.1)
Combination with cross-sectional OCTA	32 (44.4)	40 (55.6)	13 (18.1)	3 (4.2)
SD-OCTA Spectralis				
Avascular complex slab	19 (26.4)	53 (73.6)	14 (19.4)	9 (12.5)
Manual slab	20 (27.8)	52 (72.2)	15 (20.8)	13 (18.1)
Combination with cross-sectional OCTA	33 (45.8)	39 (54.2)	13 (18.1)	3 (4.2)

*MNV* macular neovascularization, *FA* fluorescein angiography, *ORCC* outer retina to choriocapillaris, *SD-OCTA* spectral domain angiography, *SS-OCTA* swept-source angiography

**Table 4:** Sensitivity, specificity, and concordance compared to FA for SS-OCTA and SD-OCTA

	SEN (%)	SPE (%)	Concordance to FA (Cohen’s kappa (% agreement))	Q *n* (%)	CG *n* (%)
SS-OCTA PLEX Elite 9000					
ORCC slab	51.7	93.0	0.48 (76.4)	15 (20.8)	2 (2.8)
Manual Slab	79.3	86.0	0.65 (83.3)	7 (9.7)	0
Combination with cross-sectional OCTA	82.8	81.4	0.63 (81.9)	3 (4.2)	0
SD-OCTA Spectralis					
Avascular complex slab	58.6	95.3	0.57 (80.5)	7 (9.7)	3 (4.2)
Manual slab	58.6	93.0	0.62 (79.2)	11 (15.3)	1 (1.4)
Combination with cross-sectional OCTA	86.2	81.4	0.66 (83.3)	3 (7.2)	0

*CG* grading value “cannot grade”, *ORCC* outer retina to choriocapillaris, *Q* grading value “questionable”, *SEN* sensitivity, *SPE* specificity, *SS-OCTA* swept-source optical coherence tomography angiography.

Both devices reached similar results regarding sensitivity and specificity for the detection of MNV, regarding the number of cases with discrepancies to FA and the number of cases evaluated as “questionable” or “cannot grade” indicating less confidence in image interpretation or poor image quality. Concordance to FA was lowest for the automated slabs for both devices. Discrepancies between devices were lowest when evaluating the combination of en face OCTA and cross-sectional OCTA with only 3 out of 72 eyes.

Fleiss kappa as measure of concordance between FA as ground truth and SS-OCTA and SD-OCTA was 0.56 (moderate) for the automated slabs, 0.60 (moderate) for the manual slabs, and increased to 0.73 (good agreement) for the combination of en face OCTA with cross-sectional OCTA.

## Discussion

MNV might occur as a complication of various chorioretinal diseases. While FA has been the gold standard for MNV detection over decades, OCTA provides a valuable addition to multimodal imaging as it allows non-invasive visualization and quantification of the retinal and choroidal vasculature, combining flow information and structural OCT [5, 7, 8]. Reported sensitivity and specificity values for identification of MNV with OCTA largely vary in the literature [9–12]. Studies differ regarding patient characteristics, OCTA devices, as well as imaging and analysis methods used. Our study is the first to directly compare the PLEX Elite SS-OCTA with the Spectralis SD-OCTA device for the detection of MNV in patients with various chorioretinal diseases using three different approaches including analysis of automated en face OCTA alone, manually modified en face OCTA alone and the combination of en face OCTA with cross-sectional OCTA.

Our results indicate that overall both the PLEX Elite SS-OCTA and the Spectralis SD-OCTA device achieve similar sensitivity and specificity values for MNV detection. Identification of MNV only based on preset en face OCTA slabs was limited with both devices. This may be explained by artifacts on OCTA en face images caused by e.g. segmentation errors, which may impede image evaluation [1, 13, 14]. A total of 21% of PLEX Elite SS-OCTA scans and 10% of Spectralis SD-OCTA scans were evaluated as “questionable” for presence of MNV, and 3%, respectively 4% were considered “cannot grade” when using preset en face slabs. This reflects a certain insecurity of graders to identify a MNV lesion on en face images with potential artifacts without further information about the underlying disease, size and location of the MNV.

In our study, we created a manual slab in addition to the available preset slabs with automatically positioned segmentation lines. The aim of the manual slab was to improve visualization of MNV lesions by ensuring that these lesions are captured completely between the inner and outer segmentation line and that no parts of a MNV lesion were truncated. Manual drawing of segmentation lines on every single OCTA B-scan is time consuming and thus not feasible within clinical routine. Instead, we created our custom slabs by manual positioning of preset segmentation lines. For the PLEX Elite SS-OCTA device, we chose two RPE-fit segmentation lines as inner and outer boundary, as this line aims to follow the curvature of Bruch’s membrane. The Spectralis SD-OCTA device allows to select two transverse lines that we shifted anterior and posterior to any SHRM and/or PED. As a result, our manual slabs differ between both devices in that for SS-OCTA, the segmentation lines follow the retinal curvature, while they do not for SD-OCTA. This difference becomes most apparent in eyes with high myopia or when the OCTA B-scan is captured diagonally in the display window, as slabs created using transverse lines capture larger areas of the choroid compared to slabs created using a segmentation following Bruch’s membrane. This may explain our observation that sensitivity increased for manual en face OCTA slabs compared to automated en face OCTA slabs only for the PLEX Elite SS-OCTA but not for the Spectralis SD-OCTA in our dataset.

Combination of the en face view with the cross-sectional view resulted in the highest sensitivity values and lowest rate of “questionable” and “cannot grade” answers for both devices, as well as in the lowest discrepancy between devices and highest concordance between FA and both devices. This effect has been described previously [9, 11]. The availability of structural OCTA B-scans provides information about presence, size and location of SHRM and/or PEDs as suspicious areas for MNV, which may increase the confidence of graders in evaluating MNV. Further, information about the position of the automatically provided segmentation lines in preset slabs allowed the graders to better evaluate whether detectable flow information on en face images may be caused by segmentation artifacts.

Another potential explanation for differences in image interpretation between both devices is technical differences. The light source in SD-OCTA systems is a broadband near-infrared superluminescent diode with a center wavelength of 840 nm and a spectrometer as detector whereas the SS-OCT uses a tunable swept-laser with a center wavelength of 1050 nm and a single photodiode detector. Therefore, SS-OCTA allows a faster scanning speed and denser scanning patterns resulting in higher imaging quality. Furthermore, the longer wavelength in SS-OCTA allows a deeper penetration of light and improves the detection of even weaker signals coming from the deep retinal layers and the choroid and this might influence image presentation and thus grading results [4].

Some authors reported no significant differences regarding quantitative OCTA measurements of MNV when using different available SD-OCTA [15] or SS-OCTA [16] devices. Comparing SD-OCTA with SS-OCTA devices, studies found differences in quantitative measurements of MNV area, suggesting that SS-OCTA may be better able to demarcate the full extent of MNV vasculature compared to SD-OCTA [17, 18]. Other studies in healthy eyes reported no statistically significant differences when analyzing quantitative parameters with SS-OCTA versus SD-OCTA [7].

Strengths of our study include the prospective approach and the analysis of images by reading center graders. Further, we separately evaluated en face OCTA images alone as well as en face OCTA images in combination with cross-sectional OCTA B-scans blinded to all other imaging modalities. Furthermore, we aimed to optimize MNV detection by modification of preset segmentation lines. This approach is less time consuming compared to manual modification of segmentation lines in all OCTA B-scans and thus more feasible in daily clinic. A further strength of our study is the heterogeneity of underlying diseases, which closely resembles the spectrum of clinical cases seen in a routine retina clinic.

A limitation of our study is that the sample size was relatively small, thus identification of smaller discrepancies between subgroups, such as different underlying diseases, was not possible. Another limitation of our study is that the PAR function was turned off for both devices, because PAR was not yet available for the PLEX Elite SS-OCTA at the time our study was designed. Projection artifacts of retinal vessels at the level of the RPE may impede detection of MNV on OCTA en face images [1, 15]. In our study, projection artifacts may have led to false positive assessments regarding the presence of MNV.

In conclusion, our results indicate that both SS-OCTA and SD-OCTA achieve comparable results for identification of MNV lesions in eyes with various chorioretinal diseases. Discrepancies between devices in our study have been detected mostly for manually modified slabs, which may be explained by differences in segmentation lines used. Our results provide further evidence that evaluation of cross-sectional OCTA B-scans in combination with en face OCTA improves detection of MNV compared to evaluation of en face images alone.

## Data Availability

Not applicable.

## References

[1] Spaide RF, Fujimoto JG, Waheed NK (2015). Image artifacts in optical coherence tomography angiography. Retina.

[2] Spaide RF, Fujimoto JG, Waheed NK, Sadda SR, Staurenghi G (2018). Optical coherence tomography angiography. Prog Retin Eye Res.

[3] Coscas GJ, Lupidi M, Coscas F, Cagini C, Souied EH (2015). Optical coherence tomography angiography versus traditional multimodal imaging in assessing the activity of exudative age-related macular degeneration: a new diagnostic challenge. Retina.

[4] Diaz JD, Wang JC, Oellers P, Lains I, Sobrin L, Husain D, Miller JW, Vavvas DG, Miller JB (2018). Imaging the deep choroidal vasculature using spectral domain and swept source optical coherence tomography angiography. J Vitreoretin Dis.

[5] Spaide RF, Klancnik JM, Cooney MJ (2015). Retinal vascular layers imaged by fluorescein angiography and optical coherence tomography angiography. JAMA Ophthalmol.

[6] Siggel R, Spital C, Lentzsch A, Liakopoulos S (2020). Comparison of automated versus manually modified OCT angiography en face slabs for detection of choroidal neovascularization. Ophthalmol Retina.

[7] Al-Sheikh M, Falavarjani KG, Tepelus TC, Sadda SR (2017). Quantitative comparison of swept-source and spectral-domain OCT angiography in healthy eyes. Ophthalmic Surg Lasers Imaging Retina.

[8] de Carlo TE, Bonini Filho MA, Chin AT, Adhi M, Ferrara D, Baumal CR, Witkin AJ, Reichel E, Duker JS, Waheed NK (2015). Spectral-domain optical coherence tomography angiography of choroidal neovascularization. Ophthalmology.

[9] Inoue M, Jung JJ, Balaratnasingam C, Dansingani KK, Dhrami-Gavazi E, Suzuki M, de Carlo TE, Shahlaee A, Klufas MA, El Maftouhi A, Duker JS, Ho AC, Maftouhi MQ, Sarraf D, Freund KB, Group C-S (2016). A comparison between optical coherence tomography angiography and fluorescein angiography for the imaging of type 1 neovascularization. Invest Ophthalmol Vis Sci.

[10] Gong J, Yu S, Gong Y, Wang F, Sun X (2016). The Diagnostic Accuracy of Optical coherence tomography angiography for neovascular age-related macular degeneration: a comparison with fundus fluorescein angiography. J Ophthalmol.

[11] Faridi A, Jia Y, Gao SS, Huang D, Bhavsar KV, Wilson DJ, Sill A, Flaxel CJ, Hwang TS, Lauer AK, Bailey ST (2017). Sensitivity and specificity of OCT angiography to detect choroidal neovascularization. Ophthalmol Retina.

[12] Carnevali A, Cicinelli MV, Capuano V, Corvi F, Mazzaferro A, Querques L, Scorcia V, Souied EH, Bandello F, Querques G (2016). Optical coherence tomography angiography: a useful tool for diagnosis of treatment-naive quiescent choroidal neovascularization. Am J Ophthalmol.

[13] Ghasemi Falavarjani K, Al-Sheikh M, Akil H, Sadda SR (2017). Image artefacts in swept-source optical coherence tomography angiography. Br J Ophthalmol.

[14] Ferrara D (2016). Image artifacts in optical coherence tomography angiography. Clin Exp Ophthalmol.

[15] Lang GE, Enders C, Loidl M, Lang GK, Werner JU (2017). Accurate OCT-angiography interpretation-detection and exclusion of artifacts. Klin Monbl Augenheilkd.

[16] Falavarjani KG, Mehrpuya A, Amirkourjani F (2017). Effect of spectral domain optical coherence tomography image quality on macular thickness measurements and error rate. Curr Eye Res.

[17] Zhang Q, Zhang A, Lee CS, Lee AY, Rezaei KA, Roisman L, Miller A, Zheng F, Gregori G, Durbin MK, An L, Stetson PF, Rosenfeld PJ, Wang RK (2017). Projection artifact removal improves visualization and quantitation of macular neovascularization imaged by optical coherence tomography angiography. Ophthalmol Retina.

[18] Arya M, Rebhun CB, Cole ED, Sabrosa AS, Arcos-Villegas G, Louzada RN, Novais EA, Lane M, Dang S, Avila M, Witkin AJ, Baumal CR, Duker JS, Waheed NK (2019). Visualization of choroidal neovascularization using two commercially available spectral domain optical coherence tomography angiography devices. Retina.

[19] Ohayon A, Sacconi R, Semoun O, Corbelli E, Souied EH, Querques G (2020). Choroidal neovascular area and vessel density comparison between two swept-source optical coherence tomography angiography devices. Retina.

[20] Novais EA, Adhi M, Moult EM, Louzada RN, Cole ED, Husvogt L, Lee B, Dang S, Regatieri CV, Witkin AJ, Baumal CR, Hornegger J, Jayaraman V, Fujimoto JG, Duker JS, Waheed NK (2016). Choroidal neovascularization analyzed on ultrahigh-speed swept-source optical coherence tomography angiography compared to spectral-domain optical coherence tomography angiography. Am J Ophthalmol.

[21] Zhang Q, Chen CL, Chu Z, Zheng F, Miller A, Roisman L, de Oliveira R, Dias J, Yehoshua Z, Schaal KB, Feuer W, Gregori G, Kubach S, An L, Stetson PF, Durbin MK, Rosenfeld PJ, Wang RK (2017). Automated quantitation of choroidal neovascularization: a comparison study between spectral-domain and swept-source OCT angiograms. Invest Ophthalmol Vis Sci.

